# Speech Recognition of Accented Mandarin Based on Improved Conformer

**DOI:** 10.3390/s23084025

**Published:** 2023-04-16

**Authors:** Xing-Yao Yang, Shao-Dong Zhang, Rui Xiao, Jiong Yu, Zi-Yang Li

**Affiliations:** School of Software, Xinjiang University, 666, Shengli Road, Urumqi 830049, China

**Keywords:** speech recognition, spectrogram, Conformer, mandarin accent, temporal convolutional network

## Abstract

The convolution module in Conformer is capable of providing translationally invariant convolution in time and space. This is often used in Mandarin recognition tasks to address the diversity of speech signals by treating the time-frequency maps of speech signals as images. However, convolutional networks are more effective in local feature modeling, while dialect recognition tasks require the extraction of a long sequence of contextual information features; therefore, the SE-Conformer-TCN is proposed in this paper. By embedding the squeeze-excitation block into the Conformer, the interdependence between the features of channels can be explicitly modeled to enhance the model’s ability to select interrelated channels, thus increasing the weight of effective speech spectrogram features and decreasing the weight of ineffective or less effective feature maps. The multi-head self-attention and temporal convolutional network is built in parallel, in which the dilated causal convolutions module can cover the input time series by increasing the expansion factor and convolutional kernel to capture the location information implied between the sequences and enhance the model’s access to location information. Experiments on four public datasets demonstrate that the proposed model has a higher performance for the recognition of Mandarin with an accent, and the sentence error rate is reduced by 2.1% compared to the Conformer, with only 4.9% character error rate.

## 1. Introduction

In a corpus with sufficient data and in a quiet environment, current speech recognition technology can achieve good recognition results [1]. However, people’s accents are often influenced by regional dialects or their native languages. In contrast to standard Mandarin, the vowels and rhymes of pronunciation will change due to different accents, and the performance of the model will be affected as a result, with a lower recognition accuracy [2]. To address the challenge of accents, Wang et al. [3] proposed the method of maximum likelihood linear regression based on a Gaussian mixed-hidden Markov model. Using this as a basis, Zheng et al. [4] combined the maximum posterior probability and maximum likelihood linear regression methods to further improve the system performance in the study of speech recognition of Mandarin with a Shanghai accent. With the development of a deep neural network (DNN), Chen et al. [5] added an accent discrimination layer to the DNN model and recognized the specific features of the person speaking. The accent-dependent feature layer proposed by Senior et al. [6], which filters specific accent features, has promoted the research of multi-accented Mandarin speech recognition [7]. These speech recognition technologies mostly focus on modeling only one accent; this requires the integration of various modules to handle different accents when building a comprehensive speech processing system. However, China has approximately seven significant dialects, including Guanhua, Wu, Xiang, Gan, Kejia, Yue, and Min dialects, as well as several variant dialects derived from these [8]. This poses a significant challenge to speech recognition systems since they not only require specialized linguistic knowledge to classify different accents and construct corresponding pronunciation dictionaries or phoneme annotations in the modeling process, but also cannot be optimized together since different dialect recognition modules operate independently. This class of models is also known as hybrid models. As a result, hybrid dialect speech recognition systems are overly complex and difficult to train [9].

The current end-to-end speech recognition framework is based on connectionist temporal classification (CTC) and attention mechanisms; it solves the problem of joint optimization and does not require a rigorous annotation of the corpus and attention to the underlying linguistic content when performing acoustic modeling. End-to-end speech recognition research has gained significant attention among scholars and has emerged as a prominent topic in this field. In 2017, Google proposed the Transformer model [10], which uses a self-attentive mechanism instead of a long short-term memory neural network, thus enabling the better capture of long-term dependency speech feature information, Due to the advantages of convolutional neural networks (CNN) for local feature extraction, Google proposed the Conformer model in 2020; it is able to capture global as well as local features and has an improved recognition performance [11]. However, most studies on Mandarin speech recognition with accents are based on traditional speech recognition architectures. Less research has been conducted on end-to-end speech recognition frameworks.

Hence, this study aims to investigate the effectiveness of the SE-Conformer-TCN end-to-end speech recognition method in enhancing the recognition accuracy of accented Mandarin. Typically, the differences between dialects stem from variations in vowel pronunciation, such as the character “mountain” pronounced as “shan” or “san”, in different dialects. However, ignore its context, the pronunciation of “san” can imply the character “three”. Therefore, the proposed SE-Conformer-TCN method uses TCN to improve the network’s ability to handle lengthy sequences and to enable it to fully learn contextual information. Next, a squeeze-excitation block (SE block) is utilized to enhance feature representation, model dependencies for convolutional features, and calibrate vowel features via the feature recalibration capacity of SE. Additionally, the end-to-end speech recognition approach overcomes the challenge of hybrid models’ separate optimization by directly converting speech sequences to text sequences without pronunciation dictionaries or phoneme annotation, thus enabling more effective network training.

This paper’s main contributions are as follows:

(1) Embedding SE block into the Conformer model enables the network to recalibrate the extracted channel features;
(2) Without changing the parallelism of the Conformer model, temporal information is modeled using a temporal convolutional network (TCN) to enhance the acquisition of location information by the model and reduce the disappearance of location information in the post layer;
(3) A state-of-the-art performance is achieved in four public datasets, especially in terms of the character error rate.

## 2. Related Works

### 2.1. Temporal Convolutional Network

**Figure 1:**
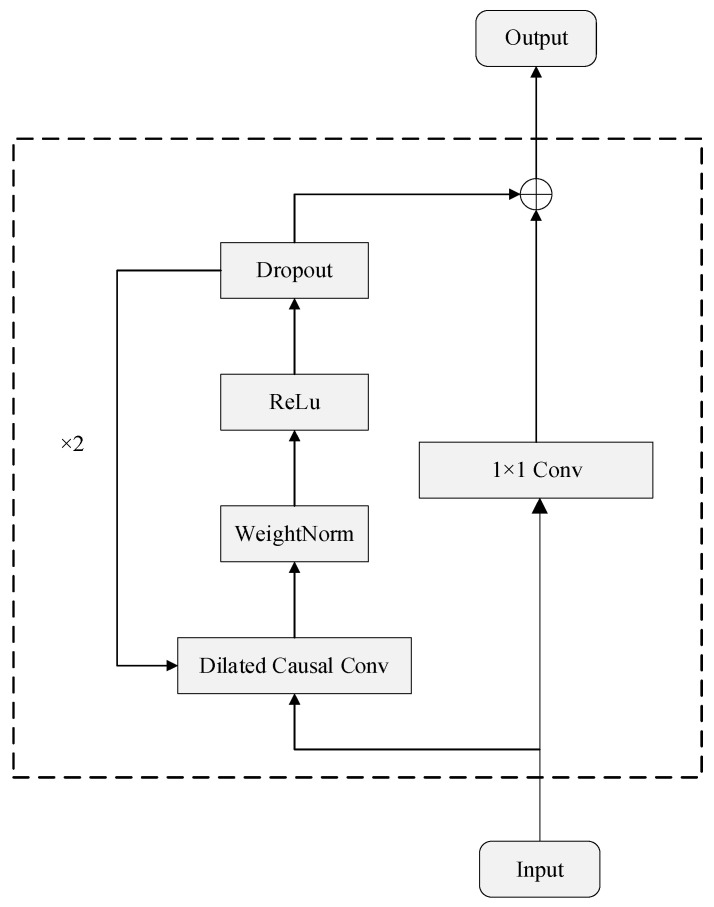
Architecture of TCN model.

TCN is a model with a unique dilation causal convolution structure based on the CNN proposed by Bai et al. [12], therefore, it is more suitable for solving temporal problems, and its structure is shown in Figure 1.

The convolution is defined as:

(1) Fs=X∗fds=∑i=0k−1fi•Xs−d·i

where the input feature sequence *X* is convolved with a dilated causal convolution kernel *f*. Here, the expansion factor *d* and convolution kernel size *k* are denoted, and s−d·i represents the corresponding location point in the input sequence. The expansion factor *d* is exponentially increased with the number of network layers *i*, which enables the TCN to effectively capture longer time series dependencies.

### 2.2. Squeeze-Excitation Block

**Figure 2:**
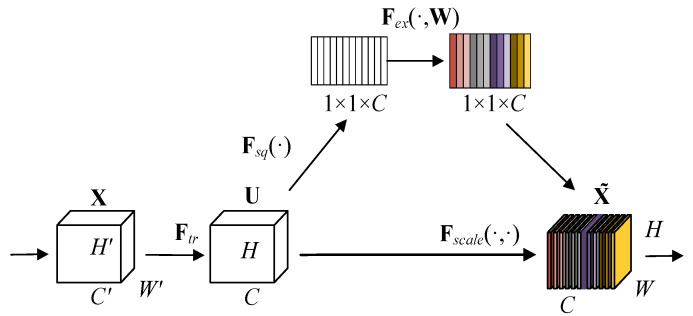
Architecture of SE Block.

The SE block proposed by Hu et al. [13] to rescale the features and its module schematic are shown in Figure 2.

The SE block recalibrates the input features in four steps as follows.

Firstly, for a convolutional operator $F_{tr}$, use $F_{tr}:X\to U,X\in {\mathbb{R}}^{H^{,}\times W^{,}\times C^{,}},U\in {\mathbb{R}}^{H\times W\times C}$ to denote the mapping process of *X* to *U*, and let $v_{c}$ refer to the parameters of the *c*-th filter. We can then write the outputs of $F_{tr}$:

(2) $$u_{c}=v_{c}*X=\sum_{s=1}^{C^{,}}v_{c}^{s}*x^{s}$$

In this equation, the variable *x* denotes the input feature vector, while *u* represents the resulting output value obtained through convolution.

The global averaging pooling operation is used in the squeeze process to compress the feature channels, and the overall ensemble operation is used to obtain a series of values with a global sensory domain. This process is calculated as follows:

(3) $$z_{c}=F_{sq}\left(u_{c}\right)=\frac{1}{H\times W}\sum_{i=1}^{H}\sum_{j=1}^{W}u_{c}\left(i,j\right)$$

where *H* and *W* can be interpreted as the spatial dimensions and $z_{c}$ represents the value obtained by compression after global average pooling.

Third, in the excitation process, the information obtained using the squeeze operation is passed through two fully connected layers, as well as the activation function, to obtain *s* of dimension 1×1×C as the weight characterizing the importance of the feature channel:

(4) $$s=F_{ex}\left(z,W\right)=\sigma \left(W_{2}\delta \left(W_{1}z\right)\right)$$

where σ refers to sigmoid function, δ represents the ReLu function, and *W* is the weight representing the feature.

Finally, the reweighting operation is performed to multiply each channel weight obtained in excitation with each of its corresponding elements, and the calculation process is shown as:

(5) $${\tilde{x}}_{c}=F_{scale}\left(u_{c},s_{c}\right)=s_{c}•u_{c}$$

where ${\tilde{x}}_{c}$ denotes the rescaled feature vector in the channel dimension.

## 3. Materials and Methods

### 3.1. Dataset

The details of the dataset are shown in Table 1.

The dataset was divided into training, testing, and validation sets without crossover, and the 80-dimensional Fbank [18] was used in all experiments. All data have a 16 kHz sample rate, 25 ms frame length, 10 ms frame shift length, and are in 16-bit monaural WAV format.

### 3.2. Experimental Environment

The experiments were conducted on Centos 7.8.2003 operating system (Centos, Raleigh, NC, USA) using Intel(R) Xeon(R) CPUE5-2640v4@2.40 GHz (Santa Clara, CA, USA) processor and a single block of NVIDIA Tesla V100 (16 GB) (Santa Clara, CA, USA) with end-to-end WeNet [19] speech recognition tool for training the model.

### 3.3. Model Construction and Speech Recognition

**Figure 3:**
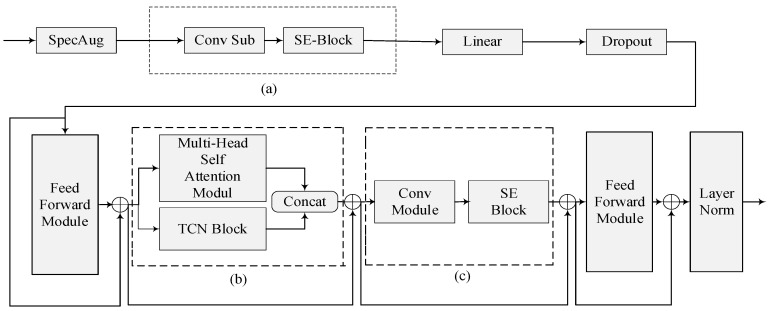
Architecture of proposed model. (**a**) First SE-Conv; (**b**) MHSA-TCN; (**c**) Second SE-Conv.

The architecture of the proposed model is illustrated in Figure 3. The SE-Conformer-TCN model consists of a SpecAug layer that enhances the input spectral features, the SE-Conv substructure (parts a and c in the figure), a linear layer, a dropout [20], and a feedforward neural network layer with a “sandwich” structure [21], an MHSA-TCN substructure (part b in the figure), a second feedforward neural network layer, and a LayerNorm [22] layer for normalization.

The main differences between the SE-Conformer-TCN model architecture and the original Conformer model are the SE-Conv and MHSA-TCN substructures.

#### 3.3.1. SE-Conv

Accurate dialect recognition requires contextual features, as the same pronunciation of a word may have different meanings depending on its context. Context-dependent features are more informative than audio features alone. In order to enhance the network’s ability to extract dependencies between channels, SE-Conv integrates the SE block into the convolution process. SE-Conv allows the network to adaptively recalibrate the importance of features between channels, with the goal of extracting more relevant contextual information, whose processing mainly comprises two phases: squeeze and excitation.

**Figure 4:**
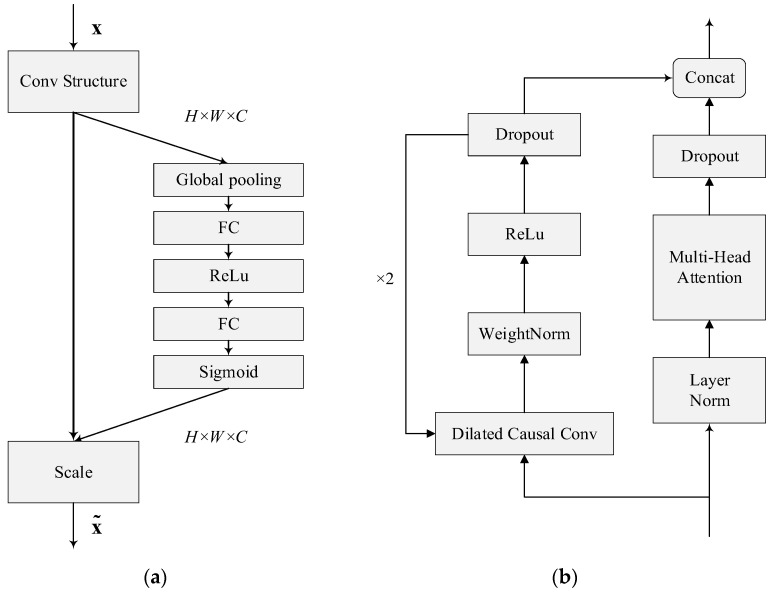
Architecture of SE-Conv and MHSA-TCN. (**a**) SE-Conv; (**b**) MHSA-TCN.

Among these, the squeeze stage is the global average pooling layer, as shown in Figure 4. We know that in the convolution process, each filter operates in a certain region, and it is difficult to obtain enough information to extract the relationship between channels, which is more significant in the front layers of the network because the perceptual field is smaller. Therefore, squeeze uses global average pooling to generate statistical information for each channel, considering it as a global feature. This process is shown in Equation (3).

The FC-ReLu-FC-Sigmoid layer shown in the figure is the excitation phase and uses a bottleneck structure containing two fully connected layers to reduce the complexity of the model and improve its generalization capability. The role of the fully connected layer is to predict the importance of each channel, obtain the weights of different channels, and apply them to the corresponding channels of the feature map. One of the purposes of using a gating mechanism with Sigmoid as well as ReLu [23] functions is to learn the nonlinear as well as nonreciprocal relationships between channels. This process is shown in Equation (4).

In summary, the SE block employs a squeeze operation to extract features from all channels, and subsequently uses an excitation operation to compute the importance weight for each feature, thereby modeling the correlation between features and selecting the relevant channels for dialect feature recalibration.

#### 3.3.2. MHSA-TCN

Attention mechanisms are currently a widely discussed topic in the field of deep learning and there exist various types of attention mechanisms, including bottom-up attention [24], global attention [25], and self-attention [26]. The concept of attention is inspired by the human visual attention mechanism, where people tend to focus on and observe a particular part of a scene instead of viewing it in its entirety [27]. By incorporating attention mechanisms into deep learning models, the network can learn to selectively focus on the most relevant information, improving its performance in various tasks [28,29,30] such as image recognition and natural language processing [31,32,33]. The attention calculation can be defined as follows:

(6) $$Attention\left(Q,K,V\right)=\mathrm{softmax}\left(\frac{QK^{T}}{\sqrt{d}}\right)V$$

where *Q* represents a query, *K* and *V* are key–value pairs, and *d* is the dimensionality of *K*. Intuitively, *Q*, *K*, and *V* represent three matrices obtained from the input data through a fully connected network. However, when the input dimension is large, the dot product will cause the amount of data to dramatically increase, and the gradient available after the softmax function is too small. Therefore, it is necessary to multiply the product of the transpose of *Q* and *K* by the inverse of the square root of *d*, and finally multiply it by the matrix *V* through the softmax layer to obtain the attention score.

Unlike the attention mechanism, TCN can control the sequence memory length by changing the size of the receptive field. Our strategy was to make full use of these two algorithms to solve our problems: the ability of the attention mechanism to select key characteristics and the ability of TCN to extract characteristics. The original TCN structure used a 1 × 1 convolutional module to perform the dimensionality reduction operation on the input of the lower layer. In order to maintain compatibility for fusing with the feature data processed by the multi-headed attention layer, the dimensionality reduction processing operation was removed in the MHSA-TCN structure. The TCN and the multi-headed self-attention were built using parallelism to process the input sequence in parallel, and thus improve the speed of the model.

The dilated causal convolution module in the MHSA-TCN structure shows an exponential growth of the expansion factor as the number of network layers increases, which can achieve better results with fewer network layers. It accelerates both the training speed and expanding perceptual domain of the model via the increase in the expansion factor; the convolution kernel can cover the input time series, and thus learn the temporal information better, fusing the features learned by this process with the features processed by the multi-headed self-attentive layer. This enhances the learning of implicit location information via the network model and extracts more features at the same time.

### 3.4. Audio Augmentation and Loss Function

**Figure 5:**
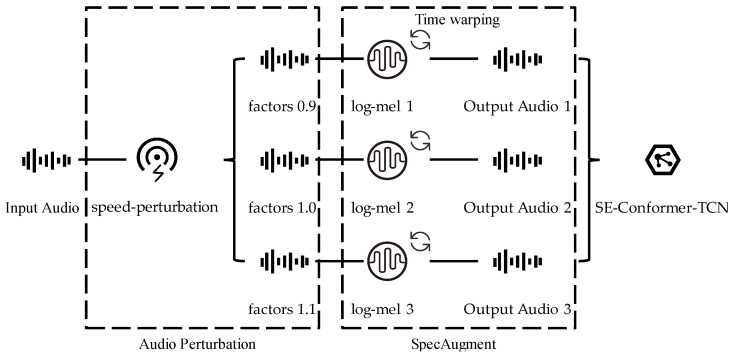
Audio Augmentation Process.

It has been shown that the use of audio perturbation [34] and SpecAugment [35] spectral data augmentation can effectively improve the performance of the model. Therefore, we conducted experiments based on these two data augmentation methods. In order to implement speed perturbation, we resampled the signal using the speed function of the Sox audio manipulation tool [36] to produce three versions of the original signal with speed factors of 0.9, 1.0, and 1.1, allowing the size of the training set to be expanded to three times the original size and to more fully utilize the data for model training. The audio was then converted into a log-mel spectrogram, and time warping and other operations that help the network learn useful features were performed on the spectrogram to make the data more robust to deformation in the time dimension. Finally, the data were converted back into audio form and fed into the model. Figure 5 shows the specific process of data augmentation.

In addition, CTC loss function is used to force the alignment of the labels [37], speed up the training of the model, and enhance the robustness of the model. For the feature vector ${\tilde{x}}_{c}$ and target sequence *y*, the CTC likelihood is defined as:

(7) $$P_{CTC}\left(y\left|{\tilde{x}}_{c}\right.\right)=\sum_{a\in \beta^{-1}\left(y\right)}P\left(a\left|{\tilde{x}}_{c}\right.\right)$$

where $\beta^{-1}\left(y\right)$ is the set of alignment, *a* to *y,* including the special blank token. The alignment probability is represented as:

(8) $$P\left(a\left|{\tilde{x}}_{c}\right.\right)=\prod_{t}P\left(a\left[t\right]\left|{\tilde{x}}_{c}\left[t\right]\right.\right)$$

where *t* denotes the *t*-th symbol of *a* and the *t*-th representation vector of ${\tilde{x}}_{c}$. At training time, we minimize the negative log-likelihood induced by CTC:

(9) $$loss_{CTC}=-\log P_{CTC}\left(y\left|x_{L}\right.\right)$$

## 4. Experiments

### 4.1. Evaluation Metrics and Hyperparameter Settings

The recognition accuracy of the model was evaluated based on character error rate (CER) and sentence error rate (SER) [38], expressed as follows:

(10) $$\begin{array}{l} CER=\frac{S+D+I}{N}\times 100\%\\ SER=\frac{Error}{Total}\times 100\% \end{array}$$

where *S* denotes the number of characters replaced, *D* refers to the number of characters deleted, *I* represents the number of characters not in the original text but inserted, and *N* is the number of all characters in the statement. *Error* in SER is the number of statements with word or character errors, and *Total* represents the number of entire statements.

Drawing on our previous experiments, we established the system parameters employed by the SE-Conformer-TCN model. The attention mechanism used a hidden layer size of 256 and had four attention heads, while the SE block employed a reduction rate of 16. The TCN network comprised three residual units with a convolutional kernel size of three and expansion factor coefficients of (one, two, three), and the filter size was set to 256. The model was trained 50 epochs and utilized a beam search strategy for decoding with a beam size of 10.

For fair comparison, we attempted to tune all the other hyperparameters to obtain the best performance for each baseline model. We repeated each experiment five times and reported the average score.

### 4.2. Baseline Preparation

Firstly, the training based on the Conformer model for the Aidatatang_200zh dataset was carried out by setting different CTC weights to derive the optimal CTC parameters to build the baseline system, and the experimental results are shown in Table 2.

Based on the Conformer model trained on the Aidatatang_200zh dataset, the model performed best when the weight size of CTC set was 0.4, achieving a character error rate of 6.2%. The performance of the model does not show a monotonic increasing or decreasing trend as the CTC weight increases, because the text labels and speech data in the manually labeled dataset are not strictly aligned. The introduced CTC enables the use of dynamic programming to cover various cases of alignment between inputs and outputs to address finer-grained alignment, thus enabling the modeling of multiple reading slices of information of a character. Then, the corresponding probability of input/output sequences is maximized by computing the loss function to improve the performance of the model. However, CTC cannot obtain the sequence global information. When the weight share of CTC increases, the gradient obtained by the self-attentive part of the model will be reduced when calculating the inverse gradient, and the model will reduce the parameter optimization of self-attentive. Therefore, when the weight of CTC was too large, the performance of the model was reduced.

In addition, we compared our method with various representative studies, including traditional speech recognition models and recent studies based on self-supervised learning.

- GMM-HMM: Phoneme recognition model based on a hidden Markov model and a hybrid Gaussian model.
- TDNN [39]: Time-delay neural networks, where phoneme-recognition-based implementation enables the network to discover acoustic-phonetic features and the temporal relationships between them independently of position in time.
- DFSMN-T [40]: Lightweight speech recognition system consisting of acoustic model DFSMN and language model transformer with fast decoding speed.
- CTC/Attention [41]: Uses a hybrid of the syllable, Chinese character, and subword as modeling units for end-to-end speech recognition system based on the CTC/attention multi-task learning.
- TCN-Transformer [42]: Transformer-based fusion of temporal convolutional neural networks and connected temporal classification.

## 5. Results

### 5.1. Recognition Results of the Model

To further illustrate the effectiveness of the proposed model architecture, the SE-Conformer-TCN model is compared with related studies in recent years in the following, and the experimental results are shown in Table 3.

It can be concluded that the experimental results based on the SE-Conformer-TCN model have performance advantages over the recent DFSMN-T, CTC/attention (character), and TCN-Transformer-CTC models, with reductions of 2.9, 1.4, and 1.3 percentage points in character error rate, respectively, confirming the validity of the proposed models. Compared with the traditional speech recognition models GMM-HMM and TDNN, the proposed model still shows better recognition of Mandarin with dialects without pronunciation dictionaries and phoneme annotation.

In order to demonstrate the generalization ability of the proposed SE-Conformer-TCN model on different datasets, this paper retrained the SE-Conformer-TCN model on the Aishell-1 dataset. To make the experiments more reliable, the model parameters were kept consistent with Conformer except for the TCN and SE block modules. The models were trained for 50 epochs, and the experimental results are shown in Table 4. 

The speech recognition system trained on the Aishell-1 dataset based on the SE-Conformer TCN model has a character error rate reduced by 1.2% compared to the Conformer, and the model architecture also has a better recognition effect on Chinese speech datasets in comprehensive domains, proving that the SE-Conformer-TCN model has a certain generalization ability.

### 5.2. Parameter Sensitivity

We conducted a series of experiments based on the CTC with a weight of 0.4 to investigate the impacts of major hyperparameters on our framework, including the reduction ratios in the SE block, and the size of TCN unit, filter, and kernel in TCN module.

The best results were achieved when the deceleration ratio size was 16, and a character error rate of 5.6% was reached.

The model performs best when the number of units of TCN is three, the filter size is 256, and the convolutional kernel size is three, achieving a character error rate of 6.0%.

Table 5 and Table 6 show the integration of the SE block and TCN into the Conformer for the best hyperparameter configuration, respectively. Both modules were integrated simultaneously and compared to the baseline model, and the experimental results are shown in Table 7.

The SE-Conformer model reduced the sentence error rate of the baseline system by about 3.1%, while the Conformer-TCN reduced it by about 1.8%, verifying the effectiveness of SE and TCN in the model. By adding both modules to the Conformer model, the final SE-Conformer-TCN model architecture achieved a significant performance improvement compared to Conformer, SE-Conformer, and Conformer-TCN, with a sentence error rate of 36.5%. This validates the proposed SE-Conformer-TCN model.

In a comprehensive analysis, the embedding of the SE block recalibrates the features extracted from the convolutional structure of the model, increases the weight of accent features in the speech data, and decreases the weight of invalid features. As a result, the model shows a significant improvement in the recognition performance of accented Mandarin. The added TCN can accurately learn the length dependence of time sequences and the location information implied by the sequences. When fused with the features processed by the multi-headed self-attentive machine layer, the model can obtain more features and location information; this is beneficial for improving the model’s performance. However, it can also be seen from the experiments that the TCN has less effect on the model’s performance improvement compared to the SE because the TCN has limitations in acquiring more position information since the multi-headed self-attentive layer in the model already captures the relative position information of sequences.

**Figure 6:**
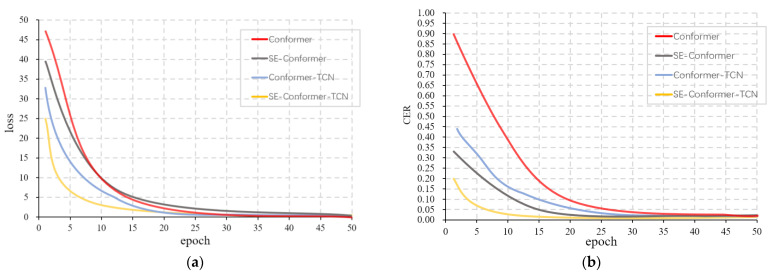
Convergence process for four experimental models. (**a**) Loss convergence process; (**b**) CER convergence process.

As can be seen in Figure 6, CTC can effectively solve the problem of the model being difficult to converge due to the different speech speeds or different lengths of time between words, thus speeding up the training speed of the model and making it possible to train the model with fewer epochs to achieve better results. We can also see that the loss of the model changes faster with the addition of the SE block and TCN, and the CER during training is lower compared with other models, because the SE block and TCN are more accurate in extracting the features of speech sequences with accents and more sensitive to capturing the location information in the sequences, which makes the model more efficient in training each epoch than the Conformer model. This demonstrates that the SE-Conformer-TCN model is effective in improving the recognition performance of accented Mandarin speech.

## 6. Conclusions

In this paper, we propose a novel approach for accented Mandarin speech recognition that utilizes a combination of a temporal convolutional network and squeeze-excitation block. The motivation behind our approach was to tackle the challenge of dialect recognition based on end-to-end model, which may significantly affect the accuracy and robustness of speech recognition systems.

To this end, our proposed model can recalibrate the features extracted from the convolutional structure, increase the weight of accent features, and decrease the weight of useless features in speech data. Additionally, the TCN can also better learn the location information implied in the sequences, which achieves a good recognition of Mandarin without extensive knowledge of accents and acoustics, while ensuring a simple and efficient network structure compared to traditional recognition methods. To evaluate the effectiveness of our method, we conducted experiments on four public datasets: Ai-datatang_200zh, Aishell-1, CASIA-1, and CASIA-2.

The experimental results show that our method achieved different degrees of improvement for these datasets, demonstrating its accuracy and robustness in speech recognition of accented Mandarin. Furthermore, we performed in-depth comparison experiments on the hyperparameter settings of various modules within the proposed model to confirm the effectiveness of each module in enhancing the performance of Conformer for dialect recognition. Specifically, our approach outperformed state-of-the-art methods in terms of multiple evaluation metrics, such as CER and SER. These results validate the effectiveness of our approach in handling challenging scenarios.

## Figures and Tables

**Table 1:** Information about datasets.

Dataset	Hour-Long	Male Speaker	Female Speaker	Speaker Regions	Sentences
Aidatatang [14]	200	299	301	34	150,000
Aishell-1 [15]	178	188	212	12	120,098
CASIA-1 [16]	80	205	202	8	100,000
CASIA-2 [17]	55	150	150	9	75,000

**Table 2:** Different CTC weights for Conformer settings.

CTC Weights	SER (%)	CER (%)
0.1	39.9	6.9
0.2	41.6	7.5
0.3	39.7	6.8
**0.4**	**38.6**	**6.2**
0.5	39.1	6.6
0.6	41.1	7.0
0.7	40.9	6.7
0.8	42.1	7.6
0.9	42.5	7.7

**Table 3:** SE-Conformer-TCN comparison with recent work.

Dataset	Metric	GMM-HMM	TDNN	DFSMN-T	CTC/Attention	TCN-Transformer	SE-Conformer-TCN
Aidatatang_200zh	CER	12.3	7.4	7.8	6.3	6.2	**4.9**
	SER	43.2	39.6	38.7	37.1	37.5	**36.5**
CASIA-1	CER	15.8	8.1	7.9	6.3	6.8	**5.6**
	SER	47.1	39.9	40.1	37.3	38.2	**37.3**
CASIA-2	CER	16.6	8.2	8.4	7.2	**6.9**	7.1
	SER	46.7	40.3	40.7	38.3	37.8	**37.2**

**Table 4:** Experimental results on dataset Aishell-1.

Dataset	Model	SER (%)	CER (%)
Aishell-1	Conformer(baseline)	37.4	5.7
	**SE-Conformer-TCN**	**35.3**	**4.5**

**Table 5:** Different reduction ratios of SE-Conformer settings.

Reduction Ratios	SER (%)	CER (%)
4	37.9	5.7
8	37.6	5.8
**16**	**37.4**	**5.6**
32	38.3	6.0

**Table 6:** Different hyperparameter settings for Conformer-TCN.

Param	Size	SER (%)	CER (%)
TCN Unit	1	40.7	6.3
	2	38.5	6.0
	**3**	**38.3**	**6.0**
	4	39.1	6.2
Filter	128	40.6	6.1
	**256**	**38.3**	**6.0**
Kernel	**3**	**38.3**	**6.0**
	5	39.6	6.1
	7	40.3	6.1

**Table 7:** Performance of Conformer with different modules.

Dataset	Model	SER (%)	CER (%)
Aidatatang_200zh	Conformer(baseline)	38.6	6.2
	SE-Conformer	37.4	5.6
	Conformer-TCN	37.9	6.0
	**SE-Conformer-TCN**	**36.5**	**4.9**

## Data Availability

This study did not report any data. We used public data for research.
